# Progress in rational methods of cryoprotection in macromolecular crystallography

**DOI:** 10.1107/S090744490903995X

**Published:** 2010-03-24

**Authors:** Thomas Alcorn, Douglas H. Juers

**Affiliations:** a Program in Biochemistry, Biophysics and Molecular Biology, Whitman College, Walla Walla, WA 99362, USA; b Department of Physics, Whitman College, Walla Walla, WA 99362, USA

**Keywords:** cryoprotection, cryosolutions, thermal contraction, crystal damage, domain structure

## Abstract

Cryogenic cooling of macromolecular crystals is commonly used for X-ray data collection both to reduce crystal damage from radiation and to gather functional information by cryogenically trapping intermediates. However, the cooling process can damage the crystals. Limiting cooling-induced crystal damage often requires cryoprotection strategies, which can involve substantial screening of solution conditions and cooling protocols. Here, recent developments directed towards rational methods for cryoprotection are described. Crystal damage is described in the context of the temperature response of the crystal as a thermodynamic system. As such, the internal and external parts of the crystal typically have different cryoprotection requirements. A key physical parameter, the thermal contraction, of 26 different cryoprotective solutions was measured between 294 and 72 K. The range of contractions was 2–13%, with the more polar cryosolutions contracting less. The potential uses of these results in the development of cryocooling conditions, as well as recent developments in determining minimum cryosolution soaking times, are discussed.

## Abstract 2

Measurements of the average thermal contractions (294→72 K) of 26 different cryosolutions are presented and discussed in conjunction with other recent advances in the rational design of protocols for cryogenic cooling in macromolecular crystallography.

## 1. Introduction

Most macromolecular crystal structures are now determined at cryogenic temperature (∼100 K) as a way of limiting the radiation damage to the crystal during the diffraction experiment (Low *et al.*, 1966 ▶; Haas & Rossmann, 1970 ▶; Dewan & Tilton, 1987 ▶; Hope, 1988 ▶). However, it is common for the cooling process to disrupt the crystal order and decrease the diffraction quality. To address this problem, the crystal is often treated with a cryoprotective solution prior to cooling (Haas & Rossmann, 1970 ▶; Hope, 1988 ▶; Henderson, 1990 ▶; Rodgers, 1994 ▶; Garman & Schneider, 1997 ▶; Schneider, 1997 ▶; Garman, 1999 ▶, 2003 ▶; Kriminski *et al.*, 2002 ▶; Juers & Matthews, 2004*b* ▶; Garman & Owen, 2006 ▶). Finding the optimal cryoprotective conditions is largely dependent on the crystal system  and often involves substantial screening. Here, we discuss recent developments in the understanding of cooling-induced damage and its prevention, with the long-term goal of reducing the trial-and-error aspect of cryoprotection.

Cooling-induced crystal damage has been described using the domain model, in which the actual imperfect crystal is composed of perfect subcrystals which may have different sizes, orientations and cell dimensions. Each of these aspects of the domain structure can become exaggerated with cooling, causing broadening of the Bragg spots (Nave, 1998 ▶; Dobrianov *et al.*, 1999 ▶; Kriminski *et al.*, 2002 ▶; Vahedi-Faridi *et al.*, 2003 ▶, 2005 ▶; Lovelace *et al.*, 2004 ▶, 2006 ▶; Jenner *et al.*, 2007 ▶; Juers *et al.*, 2007 ▶).

The underlying cause of the domain exaggerations is under investigation. In some cases, especially for large plunge-cooled crystals, temperature gradients can develop within the crystal as it is cooled (Kriminski *et al.*, 2003 ▶). If one part of the crystal undergoes its cooling-induced change before another part, defects may be produced between these two regions. Experiments show that a ‘cooling zone’ can propagate along the crystal and create a pulse of strain, possibly leaving behind a trail of exaggerated domain structure (Snell *et al.*, 2002 ▶; Lovelace *et al.*, 2006 ▶). Such inhomogeneous effects are more likely to be relevant for larger crystals and can be limited by cooling more slowly (Kriminski *et al.*, 2003 ▶), as has been observed experimentally (Yao *et al.*, 2004 ▶).

**Figure 1:**
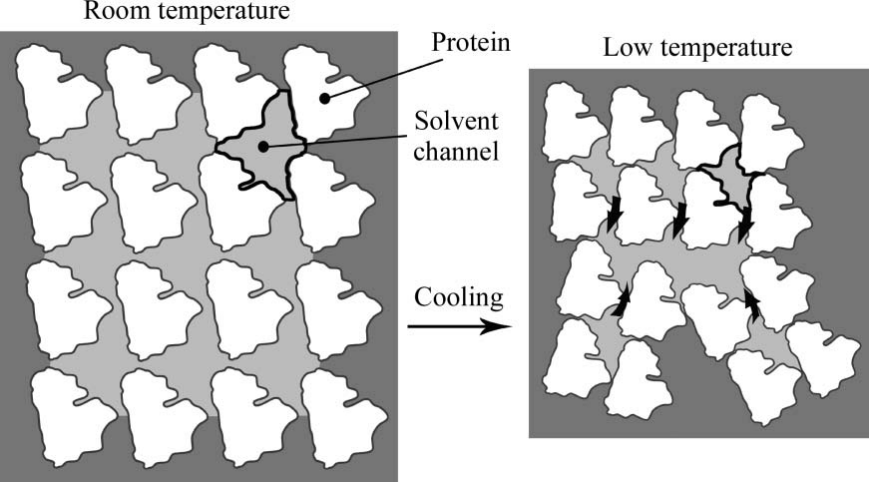
Model for cooling-induced crystal damage. Cooling triggers contraction of the protein, lattice repacking and the consequent contraction and reshaping of the solvent channel (black outline; Juers & Matthews, 2001 ▶). If the internal liquid (light gray) does not contract enough to compensate, the unit cell bursts, much as a copper pipe carrying water can burst if it is cooled below the freezing point of water. Curved black arrows show a hypothetical flow of the liquid. In this example, an idealized perfect crystal is broken into three smaller domains.

Even if the crystal is cooled uniformly without large temperature gradients, as is common for cooling in cold gas streams (Kriminski *et al.*, 2003 ▶), damage can still result. In this context, an important factor appears to be thermal contraction compensation among the different constituents of the crystal (Juers & Matthews, 2001 ▶; Kriminski *et al.*, 2002 ▶; Juers & Matthews, 2004*a* ▶,*b* ▶; Lovelace *et al.*, 2006 ▶; Fig. 1 ▶). Because the crystal is a thermodynamic system, it undergoes a response to the decrease in temperature. The macromolecules contract, as would be expected (Frauenfelder *et al.*, 1987 ▶), as do the interstices within the crystal (Juers & Matthews, 2001 ▶). Consequently, the liquid contained within the interstices must adjust or damage will result. The adjustment could take place *via* contraction as an inherent temperature response of the liquid, compression of the liquid by the contracting protein lattice or flowing of the liquid to other places in the crystal. Here, the inherent thermal contraction of the liquid will be considered.

The thermal contractions of cryosolutions have been determined for a few cases (Juers & Matthews, 2004*a* ▶). In order to achieve a more complete picture of the range of behaviors, the thermal contractions of most of the common cryosolutions used in macromolecular crystallography were measured and a range of contractions of 2–13% was found. In this paper, the cryocooling problem is discussed in the context of these and other recent results.

## 2. Materials and methods

### 2.1. Preparation of solutions

Unless otherwise noted, all chemicals were purchased from Sigma–Aldrich (St Louis, Missouri, USA). Fomblin YR-1800 was obtained from Lancaster Synthesis (Ward Hill, Massachusetts, USA). Polyethylene glycol 4000, d-sorbitol, d-(+)-xylose, polyvinylpyrrolidone K 15, lithium nitrate and sodium formate were from Fluka (Steinheim, Germany). Water was purified using an E-Pure deionization system (Barnstead International, Boston, Massachusetts, USA). Binary solutions between water and the cryoprotective agent of interest were made gravimetrically.

### 2.2. Measurement of thermal contraction

The thermal contraction was characterized using as a metric the fractional change in the specific volume (ν), 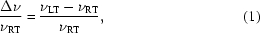
which is related to the fractional change in the density (ρ = 1/ν), 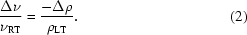
This requires density measurements of the cryosolution at the two temperatures of interest (294 and ∼77 K). Room-temperature measurements were made with either a 25 ml volumetric flask or a 1 ml pipette (VWR, West Chester, Pennsylvania, USA) calibrated with degassed deionized water at room temperature (294 K) and an electronic balance (PG503-S DeltaRange from Mettler-Toledo, Columbus, Ohio, USA). Each solution was measured 3–19 times at room temperature (highly viscous polyethylene glycol solutions required many trials) and 5–10 times at low temperature. Low-temperature measurements were made using a buoyancy-based technique with liquid nitrogen as the displaced liquid with one variation from the method previously described (Dewar, 1902 ▶; Juers & Matthews, 2001 ▶, 2004*a* ▶). Briefly, the pipette portion of a polyethylene transfer pipette (Samco Scientific, San Francisco, California, USA) was used to make an ∼0.7 ml tube with one end closed by melting. This tube was then suspended horizontally beneath a scale and its weight was measured in air and also when submerged in liquid nitrogen with and without the cryosolution in the tube. These four measurements permit the density at 77 K to be determined,
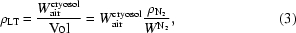
where 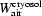 is the weight of the cryosolution in air, Vol is the volume of the cryosolution at low temperature, 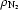 is the density of liquid nitrogen and 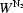 is the weight of liquid nitrogen displaced by the cryosolution.

In some cases, extra buoyancy was created from bubbles forming in the liquid nitrogen in the tube, which decreases the measured weight and causes a systematic underestimation of the density. This problem was addressed by placing the Dewar of liquid nitrogen in a bell jar attached to a vacuum pump for ∼10 min. This created supercooled nitrogen (about 67 K), which was then used as the displaced liquid, inhibiting bubble formation. This method was adopted for all solutions and the temperature of the nitrogen was measured at the same height as the tube with a platinum resistance temperature device (P/N 1PT100KN1515CLA, Omega Engineering, Inc., Stamford, Connecticut, USA; resistance was measured using a four-wire ohmmeter from Fluke Electronics, Everett, Washington, USA). This thermometer was calibrated by fitting the Callendar–Van Dusen equation describing the temperature dependence of the platinum resistance device to measurements of the resistance at four known temperatures (Childs, 2001 ▶): the melting point of nitrogen at 97.94 kPa (*T* = 63.17 K; *R* = 14.30 Ω), the boiling point of nitrogen at 96.34 kPa (*T* = 77.07 K; *R* = 20.32 Ω), the melting point of crystalline ice (*T* = 273.15 K, *R* = 100.26 Ω) and the boiling point of water (*T* = 373 K, *R* = 138.5 Ω) (Younglove, 1982 ▶). Solid nitrogen was made *via* evaporative cooling. The measured resistance was corrected for self-heating by comparing the tem­perature measured in still liquid with the temperature measured in flowing liquid. The density of the liquid at the measured temperature was then calculated from a fit of tabulated ρ *versus T* data from the nitrogen equation of state (Younglove, 1982 ▶). Because the temp­erature of the nitrogen varied between 67 and 77 K, the densities reported for these solutions may be treated as being at approximately 72 ± 5 K. The method was tested using deionized degassed water, with two different measuring tubes, yielding values of 0.932 ± 0.005  and 0.931 ± 0.003 g cm^−3^ as expected from the first buoyancy-based experiments (Dewar, 1902 ▶) and X-ray measurements on hexagonal ice (Röttger *et al.*, 1994 ▶).

### 2.3. Calculations

Solvent-accessible surface areas were determined using the program *EdPDB* (Zhang & Matthews, 1995 ▶). Ideal co­ordinates for the cryoprotective agents were downloaded from the Protein Data Bank (Berman *et al.*, 2000 ▶) and the solvent-accessible surface areas (SAS) of each of the atoms in the molecule were determined using the following radii (in Å): C = 1.8, N = 1.7, O = 1.4, S = 2.0, probe = 1.4. The polarities of the cryoprotective agent and the cryoprotective solution were calculated as SAS(N, O, S)/SAS(C, N, O, S) either for the cryoprotective agent alone or the cryoprotective agent plus an equal weight of water molecules.

The correlations between the physical characteristics of the cryoprotective agents and the solutions were calculated according to 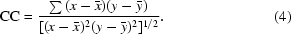
Here, *x* and *y* represent two different physical characteristics. Since not all characteristics are defined or could be found for all cryoprotective agents, the sum and averages were carried out over only the agents for which both *x* and *y* were known.

## 3. Results

Table 1 ▶ lists the measured values of the thermal contraction for 26 different cryosolutions. In all cases the concentrations used were 50%(*w*/*w*) or the solubility limit, whichever was lower. This concentration, which is relatively high for a typical macromolecular crystallography experiment, was chosen to ensure the vitrification of as many solutions as possible. Despite this, several solutions were tested that failed to vitrify completely, as indicated by opacity from small ice crystals forming in the solution during cooling. These solutions were 50%(*w*/*w*) sucrose, 50%(*w*/*w*) HEPES buffer, 25%(*w*/*w*) lithium sulfate, 25%(*w*/*w*) sodium chloride, 45%(*w*/*w*) sodium nitrate, 30%(*w*/*w*) lithium nitrate, 27%(*w*/*w*) lithium formate and 45%(*w*/*w*) d-(+)-trehalose. The expected contraction of some of these solutions upon full vitrification can be interpolated from our results (see below).

## 4. Discussion

### 4.1. Cryosolutions show a range of contractions

The contractions, which range from 2 to 13%, are related to the average bulk thermal expansion coefficient, 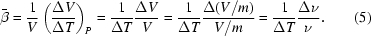
Thus, with our temperature change of Δ*T* ≃ 222 K, the range of expansion coefficients observed is 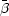 = 1–6 × 10^−4^ K^−1^. β values for some other materials are 12 × 10^−4^ K^−1^ for liquid ethanol at 298 K, 5 × 10^−4^ K^−1^ for liquid glycerol at 293 K, 2.4 × 10^−4^ K^−1^ for glassy glycerol below the glass transition at ∼180 K, 2.5 × 10^−4^ K^−1^ for water at 298 K, 1.5 × 10^−4^ K^−1^ for hexagonal ice at 250 K and 0.5 × 10^−4^ K^−1^ for copper at 298 K (Lide, 2004 ▶; Röttger *et al.*, 1994 ▶; Kauzmann, 1948 ▶). The measured contractions are thus bracketed by the values for liquid ethanol and solid ice, which is a reasonable result since on average over the whole temperature range the materials can be viewed as being between a liquid and a solid.

#### 4.1.1. Predicting the contraction of other cryosolutions

**Figure 2:**
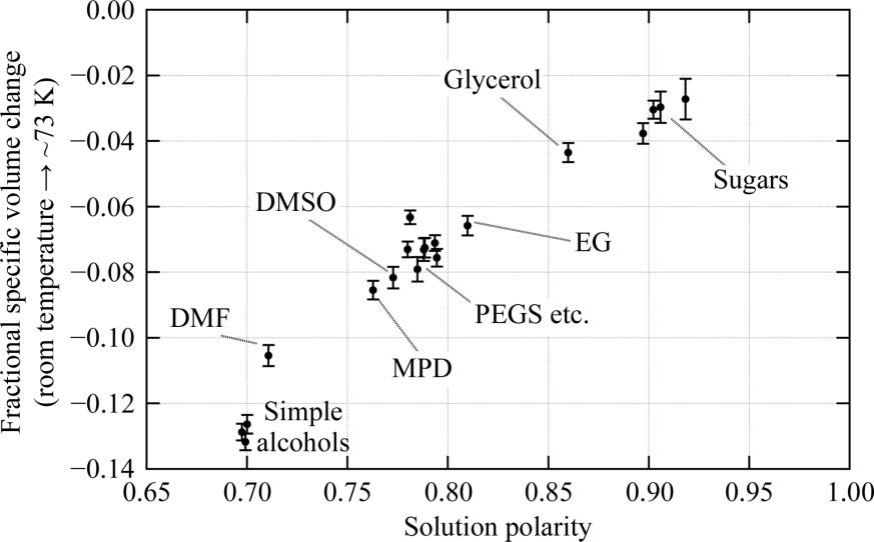
Plot of the measured thermal contraction (Δν/ν_RT_) *versus* the calculated polar surface area of the cryosolution. Data are shown for the solutions from Table 1 ▶ at 50%(*w*/*w*) cryoprotective agent/water.

To predict the contraction produced by other potential cryo­protective agents, we looked for correlations between the measured contractions and other physical characteristics of the cryoprotective agents or solutions. The results are shown in Table 2 ▶. The best correlation is with the calculated polarity of the cryosolution, which is plotted in Fig. 2 ▶. Generally, as the polarity of the solution increases its contraction decreases. Therefore, a reasonable estimation of the contractions of other potential neutral cryosolutions can probably be determined using this calculation. For example, the polarities of 50%(*w*/*w*) solutions of sucrose and trehalose are 0.92–0.93, about the same as for glucose, which indicates that the con­tractions of these solutions would be likely to be 2–3% if they vitrified. Salts and charged molecules were not included and their contractions cannot be predicted from Fig. 2 ▶. These contraction values, in combination with other recent results (Chinte *et al.*, 2005 ▶; Geremia *et al.*, 2006 ▶; Berejnov *et al.*, 2006 ▶), offer new methods of approaching the cryocooling problem.

### 4.2. Cryocooling: initial diffraction assessment

It is helpful to first assess crystal quality *via* a room-temperature diffraction measurement. While the classical capillary-mounting method can be technically challenging, especially for fragile crystals, easier methods of determining room-temperature diffraction quality have appeared in recent years. The simplest method is to use a standard cryoloop sealed by slipping it into a transparent capillary sleeve (Skrzypczak-Jankun *et al.*, 1996 ▶; Mac Sweeney & D’Arcy, 2003 ▶). This method typically requires less handling than standard capillary-mounting methods and can be carried out relatively quickly. Once the ambient temperature test has been completed, the crystal can be used for low-temperature diffraction by removing the sleeve. A version of this using thin-walled polyester tubing is commercially available (Kalinin *et al.*, 2005 ▶). For more fragile crystals, even less handling is possible by collecting room-temperature data from crystals as grown *in situ*, either in the capillary tube (Phillips, 1985 ▶) or in the crystal-growth tray. This room-temperature assessment experiment can prove very important for the following reasons. Firstly, it provides a benchmark for subsequent steps in the analysis. Poor room-temperature diffraction indicates that it may not be worth spending significant effort on finding good cooling conditions, since cooling is usually disruptive to crystal order. Good room-temperature diffraction, on the other hand, represents a target for the optimal cooling scheme. Secondly, the room-temperature measurement is needed for determination of the thermal contraction characteristics of the crystal, as discussed further below.

### 4.3. Cryocooling: choosing the cryosolution

#### 4.3.1. Internal *versus* external cryosolutions

In the model for crystal damage discussed in §[Sec sec1]1, the key role of the liquid within the crystals is to contract by the appropriate amount to compensate for the inherent contraction of the interstitial spaces (Juers & Matthews, 2001 ▶; Kriminski *et al.*, 2002 ▶; Juers & Matthews, 2004*a* ▶,*b* ▶; Lovelace *et al.*, 2006 ▶). This liquid con­traction can be modulated by adjusting the identity (Table 1 ▶ and Fig. 2 ▶) and the concentration of the cryoprotective agent.

**Figure 3:**
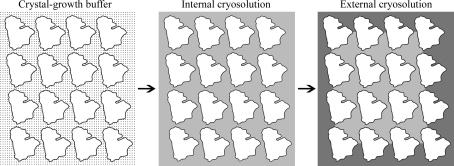
Often the crystal-growth buffer (dots) needs to be exchanged for a different solution for successful cooling. The required internal cryoprotective solution (light gray) may be different from the external cryoprotective solution (dark gray).

There is substantial evidence that the internal and external parts of the crystal may require different cryoprotection conditions. Firstly, the thermal behavior of the liquid inside the crystal depends on the diameter of the solvent channels, with smaller channels inhibiting bulk behavior (Weik *et al.*, 2001 ▶, 2005 ▶). Kwong and Liu found that in some cases the required concentration of aqueous-based cryoprotective agents could be reduced if they were used in combination with an external oil (Kwong & Liu, 1999 ▶). This suggests that lower concentrations of cryoprotective solutions are required for vitrification and cryosolution contraction within the solvent channels than when these solutions are in bulk. Secondly, because the interstitial spaces usually contract more than the unit cell (see below), the contraction needed for the liquid within the solvent channels is probably greater than the contraction needed for the external liquid. For these reasons, it is useful to consider the cryoprotection of macromolecular crystals in two phases: equilibration with an internal cryosolution followed by the replacement of the external solution with a cryo­protective buffer (Fig. 3 ▶).

#### 4.3.2. Choosing the internal cryosolution: qualitative considerations

The internal cryosolution should contain penetrating cryoprotective agents that can diffuse along the solvent channels and modulate the thermal contraction of the liquid to match the contraction of the internal spaces themselves. In the search for effective cryoprotective agents, Table 1 ▶ and Fig. 2 ▶ allow various classes of contractors to be considered. For example, sugars all give relatively low contractions, while polyethylene glycols give higher contractions. According to the model for cryo­protection described above, one would expect that if xylose is ineffective, glucose, sucrose and other sugars would also be ineffective, but that a different result might be expected with a low-molecular-weight polyethylene glycol (PEG).

It is also generally true that higher concentrations of cryoprotective agents cause greater contraction (Juers & Matthews, 2004*a* ▶), which helps to explain the observation that there can be an optimal concentration of cryoprotective agent (Mitchell & Garman, 1994 ▶). This further suggests that a lower concentration of the better contractors would be required for the same result and there is some evidence that is consistent with this idea (Juers & Matthews, 2004*b* ▶).

The choice of the internal cryosolution should also take into consideration its compatibility with the crystal. For example, many precipitants can also act as penetrating cryoprotective agents [*e.g.* 2-methyl-2,4-pentanediol (MPD), low-molecular-weight PEGs, pentaerythritol propoxylate (PEP) 426, malonate, formate and salts]. In these cases, adjusting the concentration of the precipitant may be sufficient to provide the correct thermal contraction. Larger molecules, such as high-molecular-weight PEGs, are less able to penetrate the solvent channels and often require an additional cryoprotective agent, such as a low-molecular-weight PEG. Further discussion of this topic can be found in some recent reviews (Garman, 1999 ▶; Garman & Owen, 2006 ▶).

#### 4.3.3. Choosing the internal cryosolution: a potential quantitative method

In principle, we should be able to choose the cryosolution by matching its contraction to the contraction of the solvent channel. The fractional unit-cell volume contraction [Δ_cell_ = (*V* ^lt^_cell_ − *V* ^rt^_cell_)/*V* ^rt^_cell_] may be written as a weighted sum of the contractions of the protein (Δ_prot_) and the solvent channel (Δ_ch_): Δ_cell_ = υ_prot_Δ_prot_ + (1 − υ_prot_)Δ_ch_, where υ_prot_ is the volume fraction of the unit cell occupied by the protein. This yields
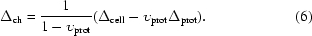
Δ_cell_ can be determined by making diffraction measurements at room and low temperature, which of course requires some initial success with cryocooling the crystal. υ_prot_ can be determined from the unit-cell parameters, space group and the molecular weight of the macromolecule (υ_prot_ ≃ 1.23/*V*_M_, where *V*_M_ is the crystal-packing parameter; Matthews, 1968 ▶). Using an average value of Δ_prot_ = −0.013 (Juers & Matthews, 2001 ▶), one can then estimate Δ_ch_ (the contraction of the channels). Table 1 ▶ can then be used to pick a cryosolution with the same contraction as the channels.

Using published average values for the contraction parameters (Δ_cell_ = −0.042, Δ_prot_ = −0.013, υ_prot_ = 0.47; Matthews, 1968 ▶; Juers & Matthews, 2001 ▶; Kantardjieff & Rupp, 2003 ▶), an average value of Δ_cell_ is found to be −0.068. At the same time, for a sample of 18 crystals (Juers & Matthews, 2001 ▶) the range of Δ_cell_ was between +0.034 and −0.151. From Table 1 ▶, this suggests that the optimal cryosolution for the average protein crystal is 50%(*w*/*w*) ethylene glycol, but also that a variety of cryosolutions will be required to optimally cool all protein crystals.

It is interesting to note that the cryosolution based on glycerol, the most commonly used cryoprotective agent (Garman & Doublié, 2003 ▶), appears in the lowest fifth of the measured contraction range. This suggests that if another cryoprotective agent, such as ethylene glycol, was adopted as the first choice rather than glycerol, lower concentrations would be necessary. Ethylene glycol is also a little smaller than glycerol and is less viscous: thus it is easier to work with and, based on its diffusion constant, should equilibrate at least 15% faster than glycerol.

Here, several assumptions are made: the unit-cell contraction is independent of the cryosolution, the contraction of the liquid in the actual protein crystal is unaffected by other components of the crystal buffer and the magnitude of the solvent contraction is unaffected by the confining nature of the solvent channels. Additionally, the contraction measurements were carried out by cooling to ∼72 K, whereas most crystals are cooled to ∼100 K for X-ray measurements. This means that the values in Table 1 ▶ are probably an overestimation of the contraction when cooling to ∼100 K, but this is likely to be by less than 10% since the extra cooling between 72 and 100 K occurs in the already vitrified state, where the contraction is lower than in the liquid state.

It remains to be determined whether or not this is a generally effective method of choosing the cryosolution. Empirical results from an earlier study using crystals of thermolysin and β-galactosidase implied that the required bulk value of the contraction may be somewhat smaller than Δ_ch_ (Juers & Matthews, 2004*a* ▶), which suggests that the predicted cryo­solution should be used at a lower concentration than 50%(*w*/*w*). This would be helpful since most crystals probably will not tolerate such high concentrations of cryoprotective agents. In any case, the findings presented here should help in the optimization of the cryoprotective solution and with the further development of predictive methods of cryoprotective agent selection.

We further note that the list of cryosolutions in Table 1 ▶ is not exhaustive. Because the cryosolution properties may be modulated inside the solvent channels and the critical con­centration for vitrification should be lower (Berejnov *et al.*, 2006 ▶) than in the ∼1 ml samples used for the contraction measurements, solutions that did not vitrify during these measurements can still be effective penetrating cryoprotective agents. Their expected contractions can be estimated by calculating the polar surface area of the solution (*e.g.* based on this idea, sucrose and trehalose solutions are expected to cause contractions similar to the glucose solution).

#### 4.3.4. Choosing the external cryosolution

The external cryosolution need not contain penetrating cryoprotective agents, but should prevent ice formation. Critical concentrations for ice prevention in crystal-sized samples of various solutions have been reported (Garman & Mitchell, 1996 ▶; McFerrin & Snell, 2002 ▶; Chinte *et al.*, 2005 ▶; Berejnov *et al.*, 2006 ▶). Thus, if the internal cryosolution is above this con­centration, it will probably be an effective external cryosolution as well. Another option, which may be especially useful in cases where the optimal internal cryosolution has a relatively low concentration of cryoprotective agent, is to use an oil, such as Paratone N, NVH oil, mineral oil or a perfluoro­polyether (Hope, 1988 ▶; Riboldi-Tunnicliffe & Hilgenfeld, 1999 ▶). This can be employed to eliminate bulk water on the outside of the crystal without affecting the composition of the internal cryosolution. In either case it seems to be helpful to remove as much of the external solution as possible prior to transferring to the cold stream.

In some cases involving very fragile crystals a relatively large volume of cryosolution is needed to support the crystal. Although no studies have been reported, in these cases some consideration may need to be given to the forces exerted on the crystal from the contracting external cryosolution and it may therefore be helpful to screen a variety of cryosolutions and concentrations, with the idea being to test a range of contractions of the external cryosolution. If the internal cryosolution is chosen correctly then the model above predicts that the external cryosolution should be chosen so that (Δν/ν_RT_) = Δ_cell_.

### 4.4. Cooling: equilibrating the crystal

Macromolecular crystals show a wide range of robustness to changes in pH, dielectric constant, ionic strength and osmotic pressure that can be encountered during equilibration with the cryosolution. For extremely sensitive crystals, growth under oil or *via* dialysis can permit equilibration with cryosolutions without exposure to air (Darcy *et al.*, 1996 ▶; Fernandez *et al.*, 2000 ▶).

If the osmotic pressure of the liquid inside the crystal and the cryosolution are different, then there will be a net flow of liquid into or out of the crystal, which can cause damage. Limiting this flow can help to preserve crystal order and yield a lower mosaicity. Some strategies to reduce this flow are to equilibrate using serial soaks (Garman, 1999 ▶), equilibrate *via* vapor diffusion (David & Burley, 1991 ▶; Wierenga *et al.*, 1992 ▶) and to adjust the composition of the cryobuffer so the osmotic pressures are identical (David, 1999 ▶). In all cases, an important question to consider is the time necessary for full equilibration with the cryosolution.

Diffusion times for small molecules through protein crystals have been measured in several different cases. For example, diffusion of glycerol into the active sites in galectin-3 crystals was recently determined to take less than 1 min (Collins *et al.*, 2007 ▶), while pyrenesulfonate took about 10 h to diffuse through lysozyme crystals (Velev *et al.*, 2000 ▶). Recently, the experiments measuring diffusion of small molecules through protein crystals were nicely summarized and simulated using a diffusion model that accounts for the protein crystal geometry (Geremia *et al.*, 2006 ▶).

The diffusion times depend on the size of the cryoprotective molecule, the solvent content of the crystal and the size of the solvent channels. Using software made available in the public domain by Geremia and coworkers, we calculated the times needed for crystals of thermolysin to equilibrate with cryosolutions of ethanol and sucrose (among the smallest and largest of the penetrating cryoprotective agents from Table 1 ▶; Geremia *et al.*, 2006 ▶). These crystals require penetrating cryoprotective agents for successful cooling, are about 50%(*v*/*v*) solvent and have tube-shaped channels that are ∼20 Å in diameter. The calculated times varied from approximately 10 s to 10 min and are summarized in Table 3 ▶.

This suggests that with some crystal/cryoprotective agent combinations soaking times will need to be several minutes or longer for full equilibration. If shorter soaks are initially tried without success, it may be helpful to increase the soaking time. On the other hand, equilibration with the external cryosolution requires no diffusion through the solvent channels and can be accomplished in less than a few seconds.

### 4.5. Other considerations

Here, we have mainly discussed the thermal contraction of the cryosolution. More precisely, we have considered the average thermal expansion, 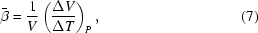
over the temperature range ∼294→∼100 K. Ideally, one would consider the continuous thermal expansion curve, 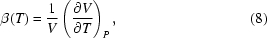
and try to design a cryosolution such that over the whole temperature range the thermal expansions of the cryosolvent and the solvent channel are identical, or β_CS_(*T*) = β_CH_(*T*). Additionally, we have not considered some physical characteristics of the cryosolution that may have an effect on the outcome, including the compressibility, viscosity and dielectric constant. A highly compressible liquid with low viscosity will give the cryosolution the ability to respond to changes in the crystal packing as the crystal is cooled by compressing or flowing into or out of solvent reservoirs either within the crystal or external to it. This might allow for successful cooling even when β_CS_(*T*) and β_CH_(*T*) are not identical over the whole temperature range. These may be useful properties of the cryosolution to consider in the future and could, for example, form the basis of cryosolution cocktails that combine multiple cryoprotective agents to provide optimal contraction and flow properties.

## 5. Summary and conclusions

Cryocooling is a necessary step for most macromolecular crystallography projects. Progress has been made over the last few years to make cryoprotection more predictable. We have described measurements of the thermal contraction of a range of cryosolutions, which provide new information for researchers to utilize in designing cryocooling procedures. These contraction measurements have led us to propose a method for choosing the optimal cryosolution that is based on simple measurements of the crystal diffraction at room temperature and low temperature. Recent work also suggests that the required soaking times for cryosolution equilibration will depend on the crystal. Together, these advances hold the potential to improve predictive possibilities in cryoprotective agent selection and protocol design in macromolecular crystallography.

## Figures and Tables

**Table 1:** Thermal contraction characteristics of cryosolutions in order of increasing contraction

Cryoprotective agent†	Concentration‡ [%(*w*/*w*)]	ρ_RT_ (*σ*)§ (g ml^−1^)	ρ_LT_ (*σ*)§ (g ml^−1^)	ρ_liq_¶ (g ml^−1^)	MW (g mol^−1^)	Specific volume change (σ)††
Lithium acetate	30	1.123 (3)	1.148 (3)	N/A	66.0	−0.022 (3)
Glucose	50	1.223 (3)	1.257 (7)	N/A	180.2	−0.027 (6)
Xylose	50	1.218 (4)	1.256 (3)	N/A	150.1	−0.030 (4)
Sorbitol	50	1.195 (3)	1.233 (2)	N/A	182.2	−0.030 (3)
Magnesium acetate	33	1.186 (3)	1.231 (3)	N/A	142.5	−0.037 (3)
Xylitol	50	1.186 (4)	1.232 (2)	N/A	152.2	−0.038 (3)
Glycerol	50	1.130 (3)	1.181 (2)	1.257	92.1	−0.044 (3)
Sodium formate	45	1.320 (3)	1.393 (2)	N/A	68.0	−0.052 (2)
Sodium malonate	45	1.356 (3)	1.432 (4)	N/A	148.1	−0.053 (3)
Lithium chloride	40	1.266 (3)	1.340 (4)	N/A	42.4	−0.056 (4)
1,6-Hexanediol	50	0.999 (2)	1.066 (1)	N/A	118.2	−0.063 (2)
Ethylene glycol	50	1.064 (3)	1.139 (2)	1.110	62.1	−0.066 (3)
PEG 200	50	1.083 (2)	1.166 (2)	1.124	200.0	−0.071 (2)
PEG 400	50	1.085 (3)	1.170 (2)	1.125	400.0	−0.072 (3)
PEP 426	50	1.055 (2)	1.138 (3)	1.050	426.0	−0.073 (3)
2,3-Butanediol	50	1.027 (2)	1.108 (2)	0.994	90.1	−0.073 (2)
PEG 600	50	1.085 (3)	1.170 (3)	1.120	600.0	−0.073 (3)
Propylene glycol	50	1.041 (2)	1.126 (2)	1.033	76.1	−0.076 (3)
PEG 4000	50	1.083 (3)	1.176 (4)	N/A	4000.0	−0.079 (4)
DMSO	50	1.080 (2)	1.176 (3)	1.095	78.1	−0.082 (3)
DP6‡‡	100	1.072 (1)	1.169 (4)	N/A	N/A	−0.083 (3)
MPD	50	0.986 (2)	1.078 (2)	0.918	118.2	−0.085 (3)
DMF	50	1.002 (2)	1.120 (3)	0.945	73.1	−0.105 (3)
2-Propanol	50	0.909 (3)	1.041 (2)	0.783	60.1	−0.126 (3)
Methanol	50	0.907 (2)	1.041 (2)	0.787	32.0	−0.129 (3)
Ethanol	50	0.913 (2)	1.052 (2)	0.787	46.1	−0.132 (3)

† Abbreviations: PEG, polyethylene glycol; PEP, pentaerythritol propoxylate; DMSO, dimethyl sulfoxide; MPD, 2-­methyl-2,4-pentanediol; DMF, *N*,*N*-dimethylformamide.

‡ All solutions were created to be 50%(*w*/*w*) at RT or, if this was not possible, at the maximum solubility. These concentrations can be converted to other measures as follows: %(*w*/*v*) = %(*w*/*w*) × ρ_RT_ and %(*v*/*v*) = %(*w*/*w*) × (ρ_RT_/ρ_liq_).

§ RT, room temperature (294 K); LT, low temperature (liquid nitrogen at ∼72 K); 3–19 measurements were made at each temperature and σ represents the statistical variation in the measurements.

¶ Density of pure liquid at ∼298 K from the literature (Yaws, 1999 ▶) or from the manufacturer.

†† The fractional change in the specific volume with cooling, (Δν/ν_RT_) = (−Δρ/ρ_LT_); σ is the propagated uncertainty.

‡‡ DP6 is a commonly used solution for cryogenic cooling of tissues (Rabin *et al.*, 1998 ▶) and is composed of about 23%(*w*/*v*) each of DMSO and propylene glycol and 3%(*w*/*v*) glucose in a HEPES/phosphate/carbonate/KCl buffer.

**Table 2:** Correlation matrix for various physical characteristics of cryoprotective agents and solutions (Yaws, 1999 ▶; Lide, 2004 ▶) The lower matrix shows the correlation coefficient, with values higher than 0.95 in bold. The diagonal shows the number of data points for each characteristic. Not all parameters are defined or could be found for all materials. The upper matrix shows the number of data points used to calculated the coefficient. PA, polar surface area. BP, boiling point. ρ_liq_, density at 298 K. ρ_RT_, density at 294 K. *B*_liq_, thermal expansion coefficient at 298 K. *P*, vapour pressure at 298 K. MP, melting point. σ, surface tension at 298 K. MW, molecular weight. ρ_LT_, density at 72 K. *K*_OW_, octanol/water partition coefficient. η, viscosity at 298 K. *k*_liq_, thermal conductivity at 298 K. Rnd, random number.

	Δν/ν_RT_†	PA†	BP	ρ_liq_	ρ_RT_†	*B*_liq_	log *P*	MP	σ	MW‡	ρ_LT_†	PA	Log *K*_OW_	η	*k*_liq_	MW	Rnd
Δν/ν_RT_†	20	19	12	10	20	10	11	13	9	14	20	19	14	10	11	20	20
PA†	**0.97**	19	12	10	19	10	11	13	9	14	19	19	14	10	11	19	19
BP	**0.96**	0.90	12	10	12	10	11	12	9	12	12	12	12	10	11	12	12
ρ_liq_	0.95	0.94	**0.98**	10	10	10	10	10	9	10	10	10	10	10	10	10	10
ρ_RT_†	0.94	**0.96**	0.91	**0.99**	20	10	11	13	9	14	20	19	14	10	11	20	20
*B*_liq_	−0.93	−0.94	−0.93	**−0.95**	−0.92	10	10	10	9	10	10	10	10	10	10	10	10
log *P*	−0.94	−0.91	**−0.97**	−0.86	−0.80	0.93	11	11	9	11	11	11	11	10	11	11	11
MP	0.92	0.90	0.84	0.85	0.90	−0.68	−0.82	13	9	13	13	13	13	10	11	13	13
σ	0.91	0.91	0.94	**0.97**	0.93	−0.94	−0.94	0.77	9	9	9	9	9	9	9	9	9
MW‡	0.87	0.89	0.77	0.77	0.87	−0.61	−0.80	0.91	0.66	14	14	14	14	10	11	14	14
ρ_LT_†	0.86	0.89	0.83	**0.96**	**0.98**	−0.87	−0.60	0.85	0.90	0.83	20	19	14	10	11	20	20
PA	0.71	0.81	0.71	0.66	0.66	−0.80	−0.56	0.74	0.71	0.76	0.58	19	14	10	11	19	19
Log *K*_OW_	−0.69	−0.79	−0.56	−0.85	−0.85	0.87	0.25	−0.57	−0.88	−0.66	−0.90	−0.78	14	10	11	14	14
η	0.61	0.68	0.71	0.65	0.59	−0.64	−0.71	0.46	0.75	0.52	0.52	0.69	−0.55	10	10	10	10
*k*_liq_	0.58	0.65	0.61	0.69	0.60	−0.84	−0.60	0.29	0.80	0.19	0.55	0.82	−0.68	0.72	11	11	11
MW	0.00	−0.02	0.77	0.77	0.09	−0.61	−0.80	0.91	0.66	1.00	0.14	−0.37	−0.66	0.52	0.19	20	20
Rnd	−0.02	0.28	−0.07	−0.31	−0.11	0.46	−0.83	0.03	−0.63	0.18	−0.22	0.05	0.03	−0.44	−0.33	0.42	20

† Physical characteristic of the 50%(*w*/*w*) cryosolution. Otherwise, the physical characteristics of the pure cryoprotective agents were used.

‡ Excluding PEG.

**Table 3:** Calculated equilibration times (*t*_0.5_, *t*_0.9_) for the diffusion of two different cryoprotective agents through the solvent channels of thermolysin crystals (about 50% solvent with 20 Å diameter channels) *t*_0.5_ and *t*_0.9_ are the times required during a diffusion-based equilibration starting with no cryoprotective agent in the crystal for the concentration to become 50% or 90% of its final value, respectively. Methanol is the smallest cryoprotective agent studied, while sucrose is one of the largest.

	Approximate equilibration times (*t*_0.5_, *t*_0.9_)	
Crystal size	Methanol	Sucrose
Small (200 × 50 × 50 µm)	3 s, 9 s	9 s, 30 s
Large (500 × 200 × 200 µm)	40 s, 2 min	2 min, 7 min
